# The genetic association between celiac disease and risk of functional dyspepsia: A Mendelian randomization study

**DOI:** 10.1097/MD.0000000000050125

**Published:** 2026-08-14

**Authors:** Jin Tang, Rong Zhao, Zhoujun Yuan, Jiayi Li, Xiaopeng Cui

**Affiliations:** a Department of Colorectal and Anal Surgery, Shanxi Provincial People’s Hospital, Taiyuan, Shanxi, China; b School of Medicine, South China University of Technology, Guangzhou, Guangdong, China; c Department of Rheumatology, The Second Hospital of Shanxi Medical University, Taiyuan, Shanxi, China; d Key laboratory of Cellular Physiology at Shanxi Medical University, Ministry of Education, Shanxi Medical University, Taiyuan, Shanxi, China; e Oncology Center, Shanxi Provincial People’s Hospital, Taiyuan, Shanxi, China.

**Keywords:** celiac disease, functional dyspepsia, GWAS, Mendelian randomization, risk factor

## Abstract

Celiac disease (CeD) is a common autoimmune disease of the small intestine triggered by ingesting gluten-containing foods. Chronic symptoms are characteristic of functional dyspepsia (FD), including chronic epigastric pain and burning. The clinical and genetic overlap between FD and CeD is currently being investigated in a large-scale comorbidity and genetic predisposition study. In this study, causal connections between CeD and FD were evaluated using Mendelian randomization (MR) methods, including the inverse variance weighted, MR-Egger regression, and the weighted median methods. Cochran *Q* test was used to examine heterogeneity among the causal effects. MR-Egger regression indicates the average pleiotropic effect of instrumental variables. MR pleiotropy residual sum and outlier analysis was conducted to detect outlier instruments and remove pleiotropy. CeD was associated with a modestly increased risk of FD, with an estimated effect size of 3.2% (odds ratio = 1.032 [1.008–1.056], *P *= .019). The reverse MR analysis provided no significant evidence supporting a causal effect of FD on CeD risk (odds ratio = 1.173 [0.989–1.390], *P *= .099). In addition, no substantial heterogeneity or horizontal pleiotropy was detected in either the forward or reverse MR analyses. These findings highlight the need for routine FD screening among patients with CeD and indicate that optimizing CeD management may help reduce the burden of FD in this population.

## 1. Introduction

Coeliac disease (CeD) is an autoimmune disorder affecting genetically predisposed individuals, both adults and children, who develop an immune response to gluten.^[1]^ Approximately 1% of Western populations are affected by CeD, and its prevalence is increasing.^[2]^ Multiple factors influence the disease, including environmental and genetic factors, as well as immune imbalance.^[3]^ Gluten, a common ingredient in daily diets, induces small-bowel mucosal villous atrophy, crypt hyperplasia, and intraepithelial lymphocytosis, and is one of the primary environmental triggers of CeD.^[3,4]^ In addition, advances in molecular genetics have identified numerous genes closely related to the pathogenesis of CeD. For example, researchers have found that individuals with specific variants of the human leukocyte antigen (HLA) DQ2 and DQ8 genes are more likely to develop CeD.^[5]^ Immunological studies of intestinal biopsies have shown that inflammatory gluten-reactive T cells recognize gluten in the context of HLA-DQ2 or HLA-DQ8 molecules, which are present only in individuals with CeD.^[6]^

Functional dyspepsia (FD) is a common gastrointestinal disorder characterized by recurrent epigastric pain or burning.^[7]^ Affecting approximately 20% of the global population, FD imposes a significant economic burden due to healthcare costs and constraints on daily activities.^[8]^ Recent studies increasingly highlight FD as an immune-mediated disorder, emphasizing the role of inflammatory mediators, immune cells (e.g., duodenal eosinophils, intraepithelial lymphocytes, and T cells), and genetic factors. Contributing factors include gut motility dysfunction, such as impaired gastric accommodation, slow gastric emptying, and increased visceral sensitivity. Genetic studies have identified polymorphisms associated with FD, such as the GNB3 C825T allele, linked to enhanced T-cell response and gastrointestinal motility issues. Further research is needed to elucidate the detailed role of genetics in FD pathophysiology.

Several observational studies have explored the relationship between CeD and FD. In the study of Dehbozorgi et al mentioned that constipation can be the 1st or primary symptom of CeD, and is particularly common in children and some adults.^[9,10]^ Bardella et al found a significantly higher prevalence of CeD among patients with dyspepsia, emphasizing the need for screening in this group.^[11]^ The study by Sahin et al similarly indicates that the prevalence of CeD in children with functional constipation is 3-fold more than in the general population.^[12]^ Routine duodenal biopsies have been shown to help identify underlying CeD in dyspeptic patients, which might otherwise be missed with standard diagnostic approaches.^[13]^ Ford et al indicated that many CeD patients continued to experience symptoms of functional gastrointestinal disorders, including FD, even after transitioning to a gluten-free diet.^[14]^ A long-term follow-up study revealed that a significant number of CeD patients continued to report symptoms despite adherence to a gluten-free diet, suggesting that other factors contribute to persistent gastrointestinal symptoms.^[15]^ Additionally, Shahbazkhani et al explored the prevalence of nonceliac gluten sensitivity in patients with dyspepsia and found that a gluten-free diet could alleviate symptoms in a subset of these patients, indicating a potential overlap between CeD and functional dyspepsia.^[16]^ Despite these studies have supported a close relationship between CeD and FD, the causal relationship between them remains to be fully elucidated. Therefore, further investigation is warranted to strengthen the evidence supporting this association and to clarify the biological mechanisms linking CeD and FD.

MR uses germline genetic variants as proxies for an exposure and can therefore strengthen causal inference when its core assumptions are satisfied.^[17,18]^ Because alleles are fixed before disease onset, MR is less vulnerable than conventional observational analyses to many acquired confounders and to reverse temporal relationships. It also offers a practical alternative when randomized trials are infeasible or unethical for etiologic questions.^[19–21]^In the present study, we used a bidirectional 2-sample MR framework to determine whether genetic liability to CeD influences the risk of FD and whether genetic predisposition to FD, in turn, affects the risk of CeD. Summary statistics from large genome-wide association studies (GWAS) were used to estimate both directions of effect and to evaluate the robustness of the findings.

## 2. Materials and methods

### 2.1. Study design

**Figure 1.**
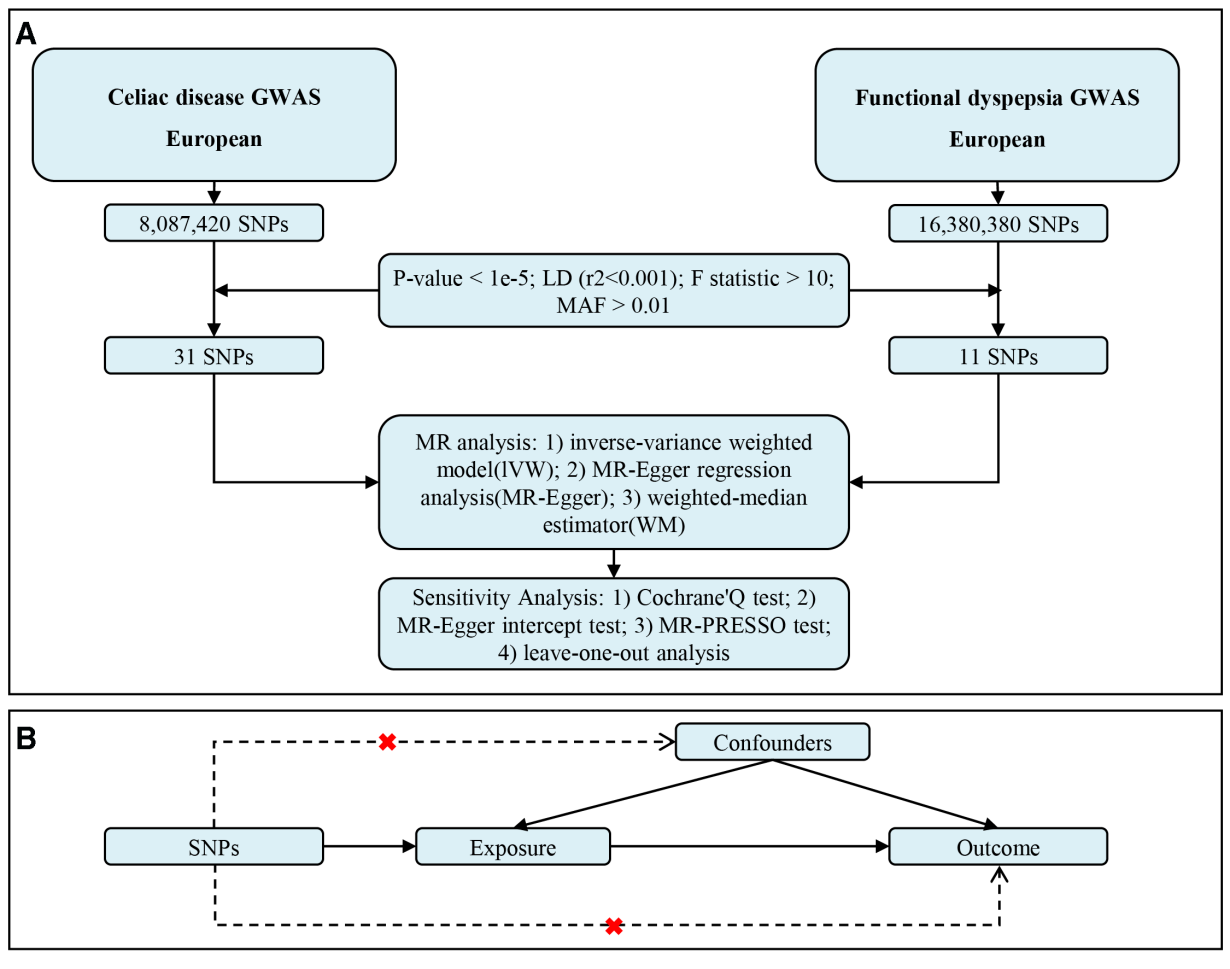
(A) Overview of the two-sample MR workflow using GWAS for celiac disease and functional dyspepsia in populations of European ancestry. (B) Schematic representation of the 3 core instrumental-variable assumptions: genetic variants are associated with the exposure, are independent of potential confounders, and affect the outcome only through the exposure. GWAS = genome-wide association study, IVW = inverse variance weighted, MAF = minor allele frequency, MR = Mendelian randomization, MR-PRESSO = Mendelian randomization pleiotropy residual sum and outlier, SNPs = single nucleotide polymorphisms.

The Figure 1A shows this study flowchart. Our study investigated the causal relationship between exposure and outcome phenotypes using MR analysis, incorporating germline genetic variations as instrumental variables (IVs). To obtain accurate results, 3 key assumptions of MR must be met (Fig. 1B): genetic IVs should be strongly associated with the exposure; genetic IVs should not be associated with exposure or outcome-related confounders; and the effect of genetic IVs on the outcome should operate solely through the exposure. Therefore, we conducted a 2-sample MR analysis to estimate the potential causal relationship between CeD and FD.

### 2.2. Data source

Based on the 1000 genomes project pilot European CEU dataset and additional resequencing studies, we compiled summary statistics for FD, including 4376 cases and 189,696 healthy controls. A total of 16,380,380 single nucleotide polymorphisms (SNPs) were included finn-b-K11_FUNCDYSP.Genetic statistics for CeD were derived from GWAS catalog (https://www.ebi.ac.uk/gwas/studies/ GCST90044158), encompassing 1036 cases and 455,312 healthy controls, all of European descent analyzed.^[22]^ These datasets utilized ICD10 hospital record codes to diagnose CeD and FD. To avoid the effects of population stratification, we only included SNPs and samples from studies involving European ancestry populations. All GWAS datasets included in this study had received approval from the relevant institutional ethics committees, and written informed consent was obtained from all participants.

### 2.3. Selection of genetic instruments

The following study filtered SNPs that met 3 key assumptions as IVs. Firstly, SNPs strongly associated with CeD were selected based on a *P* value of less than 1 × 10^−5^. Secondly, to exclude SNPs in strong linkage disequilibrium (LD), a clumping procedure was performed with an *r*^2^ < 0.001, using a clumping window size of 10,000 kb for European individuals. SNPs with a lower *P* value were retained among SNP pairings with an LD *r*^2^ greater than the threshold. Thirdly, SNPs with a minor allele frequency <0.01 were eliminated from genome-wide association studies of complex diseases. Lastly, the exposure and outcome datasets were harmonized to eliminate ambiguous SNPs with non-concordant alleles and SNPs with intermediate alleles. These chosen SNPs were utilized as the final genetic IVs for the subsequent MR analysis.^[19]^ Additionally, each selected IV was evaluated using the *F* statistic to ensure sufficient power to detect causal effects of exposure on outcomes. An *F* statistic >10 is considered powerful enough for causal effect estimation.

### 2.4. MR analysis

All analyses were performed in R software version 4.2.1 (R Foundation for Statistical Computing, Vienna, Austria) using the “TwoSampleMR” package. Before the MR analysis, the exposure and outcome datasets were harmonized by aligning effect alleles, and palindromic SNPs were excluded to reduce potential allele-orientation ambiguity. Following guideline recommendations, we primarily used the inverse variance weighted (IVW) method.^[23]^ In this approach, the Wald ratio for each SNP’s effect on the outcome was calculated, and then the results for all SNPs were combined using IVW meta-analysis. This method provides the most precise causal estimates if the MR assumptions hold true.^[24]^ To supplement the IVW results, additional methods were employed. The weighted median (WM) method offers more reliable results even when up to half of the instrumental variables (IVs) are invalid.^[25]^ Although statistically less efficient, the MR-Egger method offers an intercept estimate to test for directional pleiotropy.^[26]^ In cases of potential pleiotropy, the weighted mode method can identify valid causal effects with lower type I error rates.^[27]^

### 2.5. Sensitivity analysis

Additionally, extensive sensitivity analyses were conducted in the MR analysis to evaluate potential violations of model assumptions. Cochran *Q* test was used to assess heterogeneity across individuals. MR-Egger regression was employed to determine the average pleiotropic effect of IVs.^[28]^ Horizontal pleiotropy was further indicated by the asymmetry of the funnel plot.^[29]^ The directional horizontal pleiotropy in our MR data was examined using MR-Egger regression. To detect and correct for pleiotropy, MR pleiotropy residual sum and outlier (MR-PRESSO) analysis was performed.^[30]^ Additionally, leave-one-out analysis was utilized to assess the robustness and consistency of the results.

## 3. Results

### 3.1. Causal effects of CeD on FD

Genetic variation serves as a proxy measure for risk factors in MR to estimate the causal impact of the specified risk factor. After excluding LD (*r*^2^ < 0.001 and window size of 10,000 kb) and minor allele frequencies (effect allele frequency > 0.01), we identified 40 SNPs significantly related to CeD that were identified as IVs for subsequent MR analysis. Furthermore, 5 SNPs were excluded to meet the 3rd assumption of the MR design. These IVs had *F* statistics >10 and *r*^2^ <0.001, indicating no weak IV bias and sufficient robustness to avoid potential causality issues. After harmonizing the exposure and outcomes datasets, 4 SNPs (rs114629544, rs117088129, rs339824, and rs969021) were removed for being palindromic with intermediate allele frequencies. Finally, 31 SNPs were retained for subsequent MR analyses. Detailed information on IVs for CeD is presented in Table S1, Supplemental Digital Content 1.

**Figure 2.**
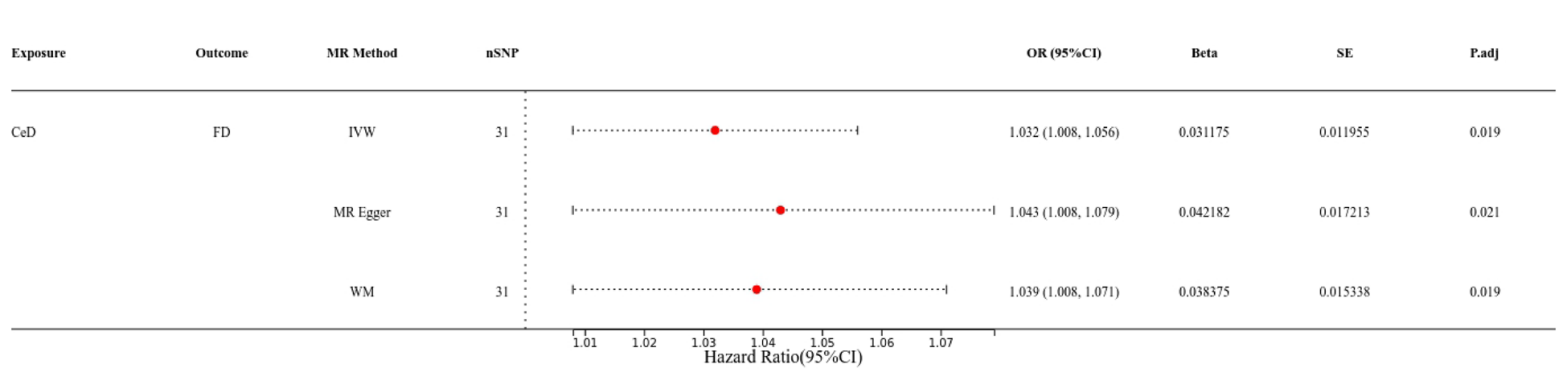
Comparison of Mendelian randomization results used by IVW, MR-Egger, and WM and visualization of forest plots. CeD = celiac disease, CI = confidence interval, FD = functional dyspepsia, IVW = inverse variance weighted, MR = Mendelian randomization, OR = odds ratio, SE = standard error, SNP = single nucleotide polymorphism, WM = weighted median.

**Figure 3.**
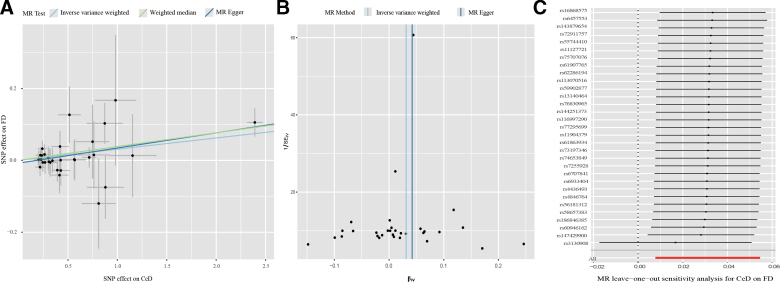
Mendelian randomization analyses of the causal effect of CeD on FD. (A) The scatter plot of MR analyses the causal effect between CeD and FD using the conventional IVW, MR-Egger, and WM methods, and each line’s slope corresponds to the expected MR effect for each method. (B) Funnel plot for assessment of horizontal pleiotropy. (C) Leave-one-out sensitivity analysis. CeD = celiac disease, FD = functional dyspepsia, IV = instrumental variables, MR = Mendelian randomization, SNP = single nucleotide polymorphism, WM = weighted median.

The preliminary analysis found a significant causal relationship between CeD and FD (IVW odds ratio [OR] = 1.032, 95% confidence interval = 1.008–1.056, *P* = .019). The outcomes of WM and ME-Egger were consistent with those of the IVW (Figs. 2 and 3A). Meanwhile, we employed the MR-Egger approach for measuring heterogeneity among the causal assessments of individual SNPs. Cochran *Q* statistic showed no substantial indication of heterogeneity between SNPs (*Q* = 14.971, *P*_*Q*_ = 0.985, Fig. 3B). Leave-one-out analysis showed the risk estimate was remarkably stable when 1 SNP was excluded (Fig. 3C). There were no significant differences in CeD and FD causal estimations, indicating that none of the discovered causative links were driven by a single IV.

### 3.2. Causal effects of FD on CeD

Nineteen FD-associated SNPs reached the predefined significance threshold and were considered as candidate instrumental variables. Following allele harmonization with the CeD outcome dataset and the exclusion of ambiguous palindromic variants, 11 independent SNPs were retained for the reverse MR analysis. The characteristics of these FD-related instruments are summarized in Table S2, Supplemental Digital Content 2.

**Figure 4.**
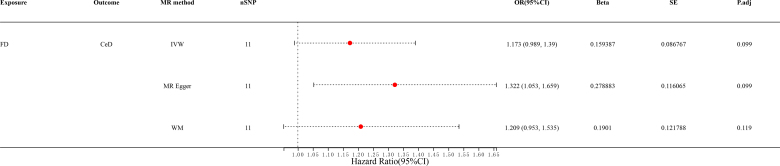
Funnel plot shows the estimation using the inverse of the standard error of the causal estimate using each SNP as a tool. The vertical line shows the results of the IVW method using all SNPs. CeD = celiac disease, CI = confidence interval, FD = functional dyspepsia, IVW = inverse variance weighted, MR = Mendelian randomization, OR = odds ratio, SE = standard error, SNPs = single nucleotide polymorphisms, WM = weighted median.

**Figure 5.**
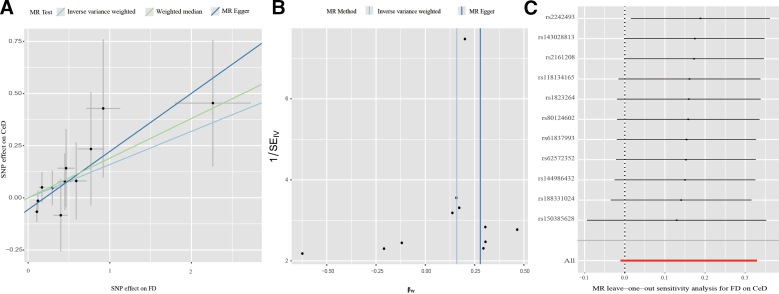
Mendelian randomization analyses of the causal effect of FD on CeD. (A) The scatter plot of MR analyses the causal effect between FD and CeD using the conventional IVW, MR-Egger, and WM methods. (B) Funnel plot for assessment of horizontal pleiotropy. (C) Leave-one-out sensitivity analysis. CeD = celiac disease, FD = functional dyspepsia, IV = instrumental variables, IVW = inverse variance weighted, MR = Mendelian randomization, SE = standard error, SNP = single nucleotide polymorphism, WM = weighted median.

Using CeD as the outcome, the IVW estimate provided no statistically significant evidence that genetically predicted FD increased the risk of CeD (OR = 1.173 [0.989–1.390], *P* = .099; Figs. 4 and 5A). The sensitivity analyses supported the stability of this result. Cochran *Q* statistic suggested little evidence of between-SNP heterogeneity (*Q* = 2.916, *P*_*Q*_ = 0.968; Fig. 5B), and the MR-Egger intercept analysis did not indicate directional pleiotropic effects. Furthermore, the leave-one-out procedure showed that the overall estimate remained stable after sequentially excluding each SNP, suggesting that the result was not disproportionately influenced by any individual variant (Fig. 5C).

## 4. Discussion

This MR analysis supports a potential causal contribution of genetic liability to CeD to the development of FD. As far as we know, this is the 1st study to assess the causal relationship between these 2 diseases using an MR design. Our findings provide genetic support for the association between CeD and FD and raise the possibility that shared biological mechanisms may contribute to their co-occurrence.

Previous observational studies have investigated the association between CeD and FD. In the study of Potter et al, they selected 3542 people from the Australian population, which found that the prevalence of symptoms consistent with functional dyspepsia in the CeD cohort was 38.9% compared to 15.6% in the non-affected population (OR 3.35, 95% confidence interval 1.72–6.52).^[31]^ Moreover, in a prospective study, Tokmak et al^[32]^ included 123 patients with CeD; among them, 78 patients were admitted to the hospital due to FD with at least an average of 3 times higher than gastroesophageal reflux disease (*P* = .044).^[32]^ At the same time, Giangreco et al showed in their study that, in patients with prolonged dyspeptic complaints, the incidence of CeD diagnosis was 2-fold higher than the incidence in the general population.^[33]^ In our research, MR analysis provided sufficient evidence to support a positive causal effect of CeD on the risk of FD, which supported these conclusions. However, the conclusions of Singh et al,^[34]^ that the prevalence of CeD in patients with dyspepsia was not significantly higher than in the general population, were contradictory to those studies and may be related to the moderate risk of selection bias and significant heterogeneity in the pooled results mentioned in their study.^[34]^

It is rationally presumed that both CeD and FD may share heritable pathogenesis based on our research. The genetic variant rs1323292 could drive altered expression of regulator of G-protein signaling-1^[35]^ and rs76830965 was lying within ILAS1-AS1 ~69 kb 5′ of IL12A on chromosome 3.^[36]^ Both of these genes are involved in the T helper cell type 1 pathway, which contributes to mucosal inflammation in active CeD^[37]^ and mediates interleukin-18 upregulation as well as activation of interleukin-1β-converting enzyme and caspase-3 to enhance mucosal injury of FDoy.^[38]^ Moreover, rs1980422, a common genetic variant of CD28, plays an important role in diverse diseases.^[39]^ Recent GWASs confirmed that CD28 was a susceptibility locus for lymphocyte and eosinophil counts.^[23]^ The number and distribution of intraepithelial lymphocytes have already been identified as key factors for the accurate diagnosis of CeD patients in previous studies.^[40–42]^ It can mediate eosinophils to upregulate serotonin release, which increases vascular permeability and promotes the occurrence of gastrointestinal diseases.^[43]^ Due to raised duodenal intraepithelial lymphocytes, chronic inflammation of the duodenum persists. Patients with FD were invariably affected by mild to moderate duodenal inflammation.^[44]^ Besides, environmental factors may also contribute to the high comorbidity rate for CeD and FD. For example, Helicobacter *pylori* might be an environmental trigger for CeD and FD.^[45–47]^ *H pylori* infection induces the infiltration of eosinophils in the mucosa and further induces the local inflammatory response.^[48]^

Our study has certain advantages. Firstly, we were able to carry out comprehensive analyses of CeD and well-powered GWAS in this study, minimizing the effect of population size. Secondly, we evaluated the relationships across 2 independent populations, and the high degree of consistency ensured that our research was robust. Additionally, sensitivity analyses were conducted to ensure the consistency and robustness of causal assessments.

There are several limitations to our study, despite these advantages. First, the research population for exposure and results had European ancestry, so applying the conclusions to populations of other ethnicities requires further study. Second, CeD is more prevalent in women than in men.^[49]^ We did not stratify the causal relationship between CeD and FD by gender due to the lack of demographic data for all GWAS participants. Third, current international diagnostic standards for CeD must be clarified.^[50]^ The inconsistent definitions of CeD used in different studies make GWASs on CeD prone to failure in identifying consistent SNPs.

In conclusion, a causal relationship exists between CeD and FD. The results suggest that CeD increases the risk of FD. Further investigation into the common underlying mechanisms between CeD and FD is necessary. Our findings provide new evidence for the prevention of FD.

## 5. Conclusions

In conclusion, a causal relationship exists between CeD and FD. The results suggest that CeD is associated with a higher risk of FD. Further investigation into the underlying common mechanisms between CeD and FD is necessary. Our findings provide new evidence for the prevention of FD. Consequently, the incorporation of diagnostic serologic assays of CeD in routine testing for dyspepsia is strongly recommended.

## Acknowledgments

This research was supported by National supercomputing Taiyuan center. And the authors thank the participants and investigators of the GWAS Catalog studies.

## Author contributions

**Data curation:** Jin Tang, Rong Zhao.

**Validation:** Zhoujun Yuan, Jiayi Li.

**Writing – original draft:** Jin Tang, Rong Zhao.

**Writing – review & editing:** Xiaopeng Cui.




