# Supplementary material for: The genetic association between celiac disease and risk of functional dyspepsia: A Mendelian randomization study
Source: Medicine (Baltimore). 2026 Aug 14;105(33):e50125. doi: 10.1097/MD.0000000000050125 (PMC13480815; doi:10.1097/MD.0000000000050125)
Supplement: Supplementary file 1 [file medi-105-e50125-s001.docx]

Supplementary table 1: Summary of the 31 SNPs in forward MR.

|  |  |  |  |  |  |  | CeD(exposure) | | | FD(outcome) | | |
| --- | --- | --- | --- | --- | --- | --- | --- | --- | --- | --- | --- | --- |
| IVs | SNP | Chr | Position | Effect allele | Other allele | F-statistic | Beta | SE | P value | Beta | SE | P value |
| 1 | rs11127721 | 3 | 81135775 | C | T | 20.16225284 | 0.217628 | 0.0484669 | 7.11E-06 | -0.0188 | -0.0188 | 0.0256 |
| 2 | rs113070516 | 2 | 49140510 | A | G | 20.28216771 | 0.26824 | 0.0595616 | 6.68E-06 | -0.0057 | -0.0057 | 0.0311 |
| 3 | rs116997290 | 8 | 60308445 | C | T | 25.41109673 | 0.568709 | 0.112818 | 4.63E-07 | 0.0011 | 0.0011 | 0.057 |
| 4 | rs11904379 | 2 | 62530788 | G | T | 19.96330627 | -0.202497 | 0.0453213 | 7.89E-06 | -0.0014 | -0.0014 | 0.0226 |
| 5 | rs13140464 | 4 | 123499745 | T | G | 33.10279211 | -0.342819 | 0.0595844 | 8.74E-09 | 8.00E-04 | 8.00E-04 | 0.0343 |
| 6 | rs143879654 | 7 | 93590971 | G | A | 22.19405628 | 0.879168 | 0.186618 | 2.46E-06 | -0.075 | -0.075 | 0.0881 |
| 7 | rs144251373 | 6 | 32088475 | A | G | 21.05889396 | -0.563795 | 0.122858 | 4.45E-06 | -0.0025 | -0.0025 | 0.0524 |
| 8 | rs147429900 | 18 | 20848668 | T | C | 22.8724544 | 0.872283 | 0.18239 | 1.73E-06 | 0.1029 | 0.1029 | 0.0567 |
| 9 | rs16868575 | 6 | 33629046 | G | A | 52.15897425 | 0.392829 | 0.0543925 | 5.12E-13 | -0.0272 | -0.0272 | 0.0321 |
| 10 | rs186846385 | 6 | 7060443 | A | C | 20.08300361 | 0.515299 | 0.114986 | 7.41E-06 | 0.1268 | 0.1268 | 0.0786 |
| 11 | rs3130908 | 6 | 31449552 | C | T | 916.0759635 | 2.3913 | 0.0790075 | 1.00E-200 | 0.1056 | 0.1056 | 0.0394 |
| 12 | rs4436493 | 10 | 52493445 | G | T | 21.80717976 | 0.235185 | 0.0503628 | 3.01E-06 | 0.0135 | 0.0135 | 0.0224 |
| 13 | rs4846764 | 1 | 222712354 | T | C | 20.6402377 | 0.221247 | 0.048699 | 5.54E-06 | 0.0143 | 0.0143 | 0.0226 |
| 14 | rs55744410 | 6 | 80117308 | C | T | 21.28100652 | 0.414068 | 0.0897585 | 3.97E-06 | -0.0411 | -0.0411 | 0.0505 |
| 15 | rs56181312 | 19 | 55900591 | A | G | 21.74104805 | 0.981817 | 0.210567 | 3.12E-06 | 0.1672 | 0.1672 | 0.1817 |
| 16 | rs58657383 | 5 | 133092934 | G | A | 22.29119912 | 0.419517 | 0.0888552 | 2.34E-06 | 0.0386 | 0.0386 | 0.0435 |
| 17 | rs59902877 | 1 | 68463905 | T | C | 21.8851945 | -0.307271 | 0.065682 | 2.89E-06 | 0.0039 | 0.0039 | 0.0348 |
| 18 | rs60946162 | 3 | 188133336 | T | C | 29.03827885 | -0.240686 | 0.0446648 | 7.10E-08 | -0.0325 | -0.0325 | 0.0223 |
| 19 | rs61863934 | 10 | 128154686 | T | C | 19.55557174 | -0.22891 | 0.0517642 | 9.77E-06 | -0.002 | -0.002 | 0.0272 |
| 20 | rs61907765 | 11 | 128391937 | T | C | 20.05913516 | 0.245655 | 0.0548491 | 7.51E-06 | -0.0057 | -0.0057 | 0.0259 |
| 21 | rs62286194 | 4 | 1620400 | A | G | 19.8414054 | 0.426072 | 0.0956526 | 8.41E-06 | 5.00E-04 | 5.00E-04 | 0.0336 |
| 22 | rs6457553 | 6 | 32330695 | G | A | 99.70535771 | -0.715217 | 0.0716273 | 1.77E-23 | -0.0082 | -0.0082 | 0.0282 |
| 23 | rs6707841 | 2 | 123822683 | G | T | 19.60920106 | 0.749172 | 0.169181 | 9.50E-06 | 0.052 | 0.052 | 0.1031 |
| 24 | rs6933404 | 6 | 137959235 | C | T | 23.51455067 | 0.26323 | 0.0542834 | 1.24E-06 | 0.0165 | 0.0165 | 0.0279 |
| 25 | rs7255928 | 19 | 40955239 | G | A | 21.32416945 | 0.204599 | 0.0443065 | 3.88E-06 | 0.0063 | 0.0063 | 0.0222 |
| 26 | rs72911757 | 1 | 55213616 | G | A | 20.38652412 | 0.427413 | 0.0946621 | 6.33E-06 | -0.0281 | -0.0281 | 0.0431 |
| 27 | rs73197346 | 21 | 36770189 | C | T | 20.61248282 | 0.300539 | 0.0661966 | 5.62E-06 | 0.0065 | 0.0065 | 0.0322 |
| 28 | rs74653849 | 14 | 87090376 | T | C | 22.46918156 | 0.762617 | 0.160884 | 2.14E-06 | 0.0151 | 0.0151 | 0.0936 |
| 29 | rs75707076 | 5 | 160179371 | C | T | 22.0273843 | 0.810811 | 0.172758 | 2.69E-06 | -0.12 | -0.12 | 0.1257 |
| 30 | rs76830965 | 3 | 159637678 | A | C | 21.21965451 | 0.321079 | 0.0697016 | 4.10E-06 | -0.0058 | -0.0058 | 0.0394 |
| 31 | rs77295699 | 12 | 1915739 | A | G | 22.72961959 | 1.15394 | 0.24204 | 1.86E-06 | 0.0133 | 0.0133 | 0.1153 |

CeD, Celiac disease; FD, functional dyspepsia; SNP, single nucleotide polymorphism; MR: Mendelian randomization; Chr, chromosome; SE, standard error; Beta, effect size (log(OR) scale) estimated with revenue for the alternative allele.
