# Supplementary material for: The genetic association between celiac disease and risk of functional dyspepsia: A Mendelian randomization study
Source: Medicine (Baltimore). 2026 Aug 14;105(33):e50125. doi: 10.1097/MD.0000000000050125 (PMC13480815; doi:10.1097/MD.0000000000050125)
Supplement: Supplementary file 2 [file medi-105-e50125-s002.docx]

Supplementary table 2: Summary of the 11 SNPs in reverse MR.

|  |  |  |  |  |  |  | FD(exposure) | | | CeD(outcome) | | |
| --- | --- | --- | --- | --- | --- | --- | --- | --- | --- | --- | --- | --- |
| IVs | SNP | Chr | Position | Effect allele | Other allele | F-statistic | Beta | SE | P value | Beta | SE | P value |
| 1 | rs118134165 | 6 | 137290259 | T | C | 20.120072 | 0.5894 | 0.1314 | 7.32E-06 | 0.080913 | 0.185116 | 0.662043 |
| 2 | rs143028813 | 13 | 75589017 | T | C | 21.461693 | 0.3998 | 0.0863 | 3.61E-06 | -0.083728 | 0.173703 | 0.629794 |
| 3 | rs144986432 | 1 | 3257010 | A | G | 22.876708 | 0.7691 | 0.1608 | 1.72E-06 | 0.234077 | 0.271297 | 0.388244 |
| 4 | rs150385628 | 10 | 121908122 | T | C | 23.539773 | 2.2619 | 0.4662 | 1.22E-06 | 0.454218 | 0.302505 | 0.133221 |
| 5 | rs1823264 | 2 | 121516307 | A | G | 20.263639 | 0.2971 | 0.066 | 6.69E-06 | 0.0469436 | 8.35E-02 | 0.57405 |
| 6 | rs188331024 | 6 | 2713397 | A | G | 19.523573 | 0.9195 | 0.2081 | 9.90E-06 | 0.428488 | 0.331897 | 0.196695 |
| 7 | rs2161208 | 5 | 85389380 | T | C | 23.994068 | -0.1205 | 0.0246 | 9.85E-07 | 0.0143747 | 0.049307 | 0.770642 |
| 8 | rs2242493 | 7 | 143028536 | A | G | 22.903061 | -0.1072 | 0.0224 | 1.62E-06 | 0.0668406 | 0.0491659 | 0.173991 |
| 9 | rs61837993 | 1 | 225995698 | A | G | 20.564042 | -0.1696 | 0.0374 | 5.77E-06 | -0.049972 | 0.0734511 | 0.496286 |
| 10 | rs62572352 | 9 | 22572193 | C | T | 20.193518 | 0.4651 | 0.1035 | 6.96E-06 | 0.141902 | 0.188289 | 0.451067 |
| 11 | rs80124602 | 3 | 117777298 | T | C | 25.714493 | 0.4503 | 0.0888 | 3.96E-07 | 0.0774048 | 0.136033 | 0.569345 |

CeD, Celiac disease; FD, functional dyspepsia; SNP, single nucleotide polymorphism; MR: Mendelian randomization; Chr, chromosome; SE, standard error; Beta, effect size (log(OR) scale) estimated with revenue for the alternative allele.
